# Comparative Effectiveness of Robotic-Assisted, Video-Assisted, and Open Thymectomy for Thymoma: A Systematic Review and Meta-Analysis

**DOI:** 10.7759/cureus.85806

**Published:** 2025-06-11

**Authors:** Quang La, Jodie Borgmann, John Tran, Francis Pryor, Jasneel S Kahlam

**Affiliations:** 1 Surgery, Futures Forward Research Institute, Toms River, USA; 2 Internal Medicine, American Preventive Screening and Education Association (APSEA), Kansas City, USA; 3 Medicine, American Preventive Screening and Education Association (APSEA), Stratford, USA; 4 Medicine, Lake Erie College of Osteopathic Medicine, Erie, USA; 5 Internal Medicine, Stony Brook Southampton, Hackettstown, USA

**Keywords:** open thymectomy, robotic, robotic-assisted thoracoscopic surgery (rats), thymectomy, video-assisted thoracoscopic surgery (vats)

## Abstract

Thymoma is the most common primary mediastinal tumor in adults, typically excised in a single operation. Less invasive approaches, specifically robotic-assisted thymectomy surgery (RATS) and video-assisted thoracoscopic surgery (VATS), have gained popularity subsequent to conventional open thymectomy via median sternotomy. To determine the best surgery for thymoma, this systematic review and meta-analysis compared RATS, VATS, and total thymectomy in terms of efficacy, safety, and prognosis. Literature databases were systematically reviewed for publications, including PubMed, Scopus, and Google Scholar, till February 2025, using the Preferred Reporting Items for Systematic Reviews and Meta-Analyses (PRISMA) criteria sectioning the articles based on comparison of RATS to VATS or open thymectomy, as well as quantitative outcomes of reoperation, total survival, recurrence, perioperative complications, length of procedure, and length of stay. The risk of bias was assessed using the Risk Of Bias In Non-randomized Studies of Interventions (ROBINS-I) tool. Eleven articles met the eligibility criteria, and data were analyzed using random-effects model meta-analysis on RevMan (Cochrane, London, UK). RATS had comparable operation times to VATS (pooled mean difference: 4.49 minutes; 95% CI: -39.87 to 48.84; I² = 98%); it was, however, linked with fewer total complications (mean difference: -3.78; 95% CI: -3.89 to -3.70) and less intraoperative blood loss (mean difference: -25.01 mL; 95% CI: -38.03 to -12.00; I² = 0%). Also, RATS showed a reduction in pleural drainage time compared to VATS (mean difference: -0.66 days; 95% CI: -0.97 to -0.35; I² = 0%). RATS led to shorter hospital stays than open thymectomy (mean difference versus VATS: -0.28 days; 95% CI: -1.36 to 0.80; I² = 91%; versus open: -1.38 days; 95% CI: -2.33 to -0.43; I² = 14%) and fewer postoperative complications than open thymectomy but not differences in oncologic outcomes, including mortality and rates of recurrence. The I² values ranged widely from 0% to 98%, indicating variable heterogeneity across outcomes, which limits interpretability in some comparisons. Most studies included were retrospective cohorts, and the risk of bias was inconsistent, with one study deemed to be at low risk and seven deemed to have some concerns. Overall heterogeneity between outcomes ranged from low to moderate (I² = 0%-98%). Issues with costs and surgeon experience variability, however, continue to be major impediments to routine use of RATS. Given the retrospective nature of most included studies and high heterogeneity in key outcomes, conclusions about RATS’s comparative advantage should be interpreted cautiously. While RATS demonstrates similar oncologic outcomes with fewer complications and shorter hospital stays, its routine use remains constrained by cost, surgeon training requirements, and a lack of high-quality prospective data. Future multicenter randomized controlled trials and cost-effectiveness studies are necessary to clarify its long-term role in thymoma surgery.

## Introduction and background

Thymoma is the most common primary mediastinal neoplasm in adults, accounting for a large proportion of anterior mediastinal neoplasms [1]. Thymomas, with an estimated incidence of 0.15 cases per 100,000 individuals, remain uncommon [2]. Thymomas are typically slow-growing with low metastatic potential, distinguishing them from thymic carcinomas, which follow a more aggressive course with poorer prognosis [3]. The majority of patients with thymoma are asymptomatic, but some present with symptoms because of compression of adjacent structures or paraneoplastic syndromes, the most frequent of which is myasthenia gravis [4]. Surgery remains the most significant and effective treatment for thymoma, offering the greatest opportunity for complete tumor resection and long-term disease control [5].

Classically, median sternotomy has been the gold standard for thymectomy, allowing direct access to the mediastinum and total resection of the thymus gland together with the surrounding fatty tissue [6]. However, while effective, open thymectomy via median sternotomy is followed by notable perioperative morbidity, prolonged hospitalization, and prolonged recovery times, prompting interest in less invasive alternatives [7].

Video-assisted thoracoscopic surgery (VATS) thymectomy was first described in the 1990s and has increasingly gained acceptance as a minimally invasive approach to open resection [7-8]. The key advantages of VATS include reduced surgical trauma, shorter hospital stay, and fewer postoperative complications. Initially conceived for benign thymic disease, VATS has been applied to malignant thymic pathology, with the literature suggesting comparable oncologic outcomes to open thymectomy [9].

Robotic-assisted thymectomy surgery (RATS) offers enhanced visualization, instrument maneuverability, and precision, particularly in the retrosternal space [10]. These advantages have contributed to the expanded application of robotic-assisted thymectomy in benign and malignant thymic pathology. Despite its potential to be advantageous, its cost, longer operative time, and need for special training are of concern [11].

Numerous reports have documented favorable results with VATS thymectomy, including reduced postoperative pain, shorter hospital stay, and fewer complications compared to open surgery [12]. More significantly, long-term survival and recurrence rates following VATS thymectomy appear comparable to those achieved with traditional open operations, making it a highly attractive option for appropriately selected patients [13]. Selection of patients, however, plays a crucial role in determining the best approach. Factors such as tumor size, location, local invasion, and myasthenia gravis symptoms can influence surgical planning, with larger or invasive tumors continuing to require open resection on a routine basis.

Despite the proliferation of minimally invasive approaches, several areas in the literature remain incomplete. Limited data are available regarding the long-term oncologic outcomes of VATS and robotic-assisted thymectomy compared to open resection [14]. Additionally, concerns regarding tumor manipulation, potential capsular rupture, and rates of local recurrence have not been fully resolved [15]. Long-term outcomes such as five-year recurrence rates, disease-free survival, and overall survival are especially underreported in the context of RATS. While randomized controlled trials are lacking, a number of recent prospective cohort studies have tried to address some of these deficits by comparing postoperative morbidity and recurrence rates between RATS and other methods, though with limited sample sizes. Because most available data are derived from non-randomized, retrospective studies, selection bias and clinical heterogeneity remain important limitations, which this review aims to address through subgroup analyses and standardized outcome comparisons.

While systematic reviews have compared minimally invasive and open thymectomy for thymic malignancies in the past, to our knowledge, no prior review has simultaneously and directly compared all three approaches, open, VATS, and RATS, within a single framework assessing shared outcomes such as complication rates, operative time, hospital stay, and oncologic efficacy. The most recent systematic review of robotic-assisted thymectomy versus video-assisted thymectomy was in 2019 [16], and there were no recent systematic reviews of robotic-assisted thymectomy versus open thymectomy. This review also incorporates more recent studies published since then, expanding the dataset and updating the comparative analysis with newly reported outcomes. A direct comparison of the three surgical methods is needed to inform clinicians in choosing the most efficacious and safest approach based on patient characteristics, available resources, and institutional expertise. It also allows structured comparison of outcomes such as complication rates, operative time, and oncologic safety between techniques.

In the current review, we perform both direct (RATS versus VATS, RATS versus open) and indirect comparisons using subgroup analyses where possible. We expect to identify trends in perioperative outcomes and determine whether minimally invasive approaches compromise oncologic efficacy. By identifying methodological gaps such as technical variability and inconsistent recurrence definitions, this study also highlights where future research should be focused. It is our goal to outline the relative strengths and weaknesses of each operative approach to inform evidence-based clinical practice.

## Review

Methods

Reporting Guidelines

**Figure 1:**
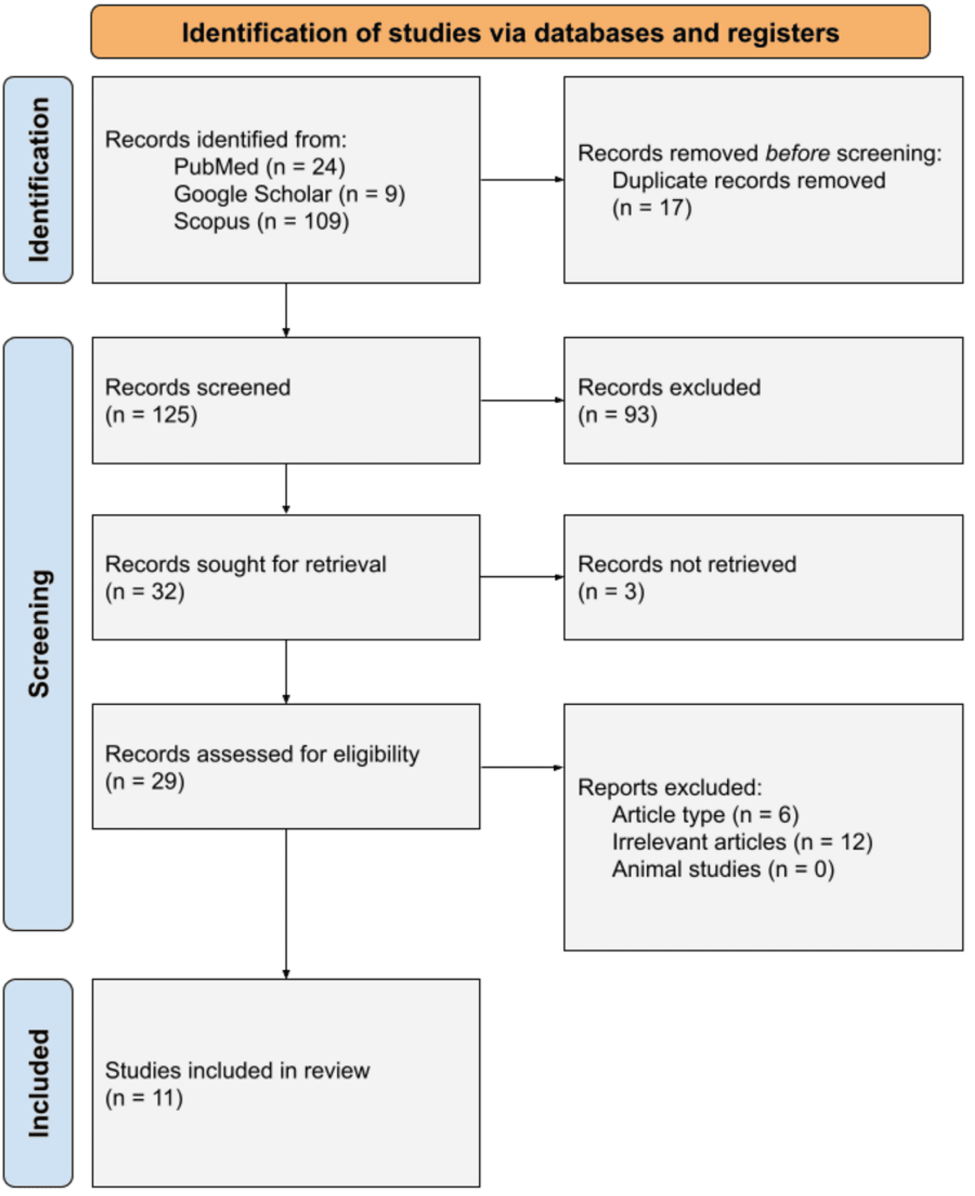
PRISMA flow diagram illustrating the screening and selection process for studies included in the analysis. PRISMA: Preferred Reporting Items for Systematic Reviews and Meta-Analyses.

This systematic review adopted the 2020 Preferred Reporting Items for Systematic Reviews and Meta-Analyses (PRISMA) guidelines to achieve methodological robustness, transparency, and reproducibility [17]. A rigorous and systematic search strategy was formulated and implemented to identify studies that studied the efficacy, safety, and complications of RATS versus VATS or open thymectomy. We searched Google Scholar, Scopus, and PubMed from the beginning of inception to 2025 using combinations of keywords relevant to thymoma, thymectomy, VATS thymectomy, robotic thymectomy, and open thymectomy. The review process included well-defined inclusion and exclusion criteria, a systematic search plan, data extraction systematically, and synthesis. Selection of studies is shown in a PRISMA flow diagram (Figure 1).

Study selection, data extraction, and risk of bias were carried out in duplicate by two reviewers (Q.L. and J.B.). Consistency was maintained with standard data extraction forms. Disagreements were resolved and discussed with a third reviewer (J.T.) when necessary.

Search Strategy

A systematic review was performed through a search of electronic databases, including PubMed, Google Scholar, and Scopus. The approach adopted a Boolean searching method using combinations of keywords and Medical Subject Headings (MeSH) terms. Keywords used in PubMed, Google Scholar, and Scopus are given in Table 1.

**Table 1:** MeSH search term strategy. MeSH: Medical Subject Headings, RATS: robotic-assisted thymectomy surgery, VATS: video-assisted thoracoscopic surgery.

Database	Search String
PubMed	("Thymoma"[Mesh] OR "Thymic Neoplasms"[Tiab] OR "Thymectomy"[Mesh] OR "Thymic Tumor*" OR "Thymic Neoplasm*" OR "Thymectomy") AND ("Robotic Surgical Procedures"[Mesh] OR "Robotic Thymectomy" OR "Robot-Assisted Surgery" OR "RATS") AND ("Thoracic Surgery, Video-Assisted"[Mesh] OR "Video-Assisted Thoracoscopic Surgery" OR "VATS") AND ("Sternotomy" OR "Open Thymectomy" OR "Median Sternotomy") AND ("Survival" OR "Recurrence" OR "Outcomes" OR "Complications" OR "Hospital Stay" OR "Postoperative Morbidity")
Scopus	TITLE-ABS-KEY ("thymoma" OR "thymic neoplasm" OR "thymectomy") AND ("robotic-assisted surgery" OR "robotic thymectomy" OR "RATS") AND ("VATS" OR "video-assisted thoracoscopic surgery") AND ("sternotomy" OR "open thymectomy" OR "median sternotomy") AND ("survival" OR "recurrence" OR "complications" OR "hospital stay" OR "morbidity")
Google Scholar	("Thymoma" OR "Thymic Neoplasm" OR "Thymectomy") AND ("Robotic Thymectomy" OR "Robotic-Assisted Surgery" OR "RATS") AND ("Video-Assisted Thoracoscopic Surgery" OR "VATS") AND ("Sternotomy" OR "Open Thymectomy" OR "Median Sternotomy") AND ("Survival" OR "Recurrence" OR "Complications" OR "Hospital Stay" OR "Morbidity")

Eligibility Criteria

Studies were selected according to pre-defined eligibility criteria including population, intervention, comparison, outcomes, and study design (Population, Intervention, Comparator, Outcome (PICO) framework). Inclusion criteria focused on English-language, peer-reviewed publications with outcomes of da Vinci robotic-assisted thymectomy in adult thymoma patients with particular comparison to VATS or open methods. Exclusion criteria removed non-comparative trials, non-primary research (e.g., reviews, editorials), non-English language literature, and studies using other methods (e.g., single-port or hand-assist methods). Exclusion criteria other than those listed included redundant datasets, no quantitative outcome data in studies, and no procedure or study arm stratification in analyses. The full eligibility criteria are presented in Table 2.

**Table 2:** Eligibility criteria for the inclusion and exclusion of studies. VATS: video-assisted thoracoscopic surgery.

Category	Inclusion Criteria	Exclusion Criteria
Population	Adult humans undergoing thymectomy for thymoma.	Studies involving non-adult populations or non-human subjects.
Intervention	Robotic-assisted thymectomy using the da Vinci system.	Studies not involving da Vinci robotic-assisted thymectomy.
Comparison	Comparative studies reporting robotic versus VATS or open thymectomy.	Studies without a comparator (robotic versus VATS or open).
Outcomes	Quantitative results for at least one outcome of interest (e.g., efficacy, safety, survival, complications).	Studies lacking quantitative results for outcomes of interest.
Study design	Primary source comparative articles (e.g., clinical trials, cohort studies).	Reviews, health technology assessments not published in peer-reviewed journals, or non-primary research.
Language	English-language publications.	Publications in languages other than English.
Publication type	Peer-reviewed journal articles.	Abstracts, books, book chapters, grey literature, or unpublished data.
Technique	Standard robotic, VATS, or open thymectomy approaches.	Alternative techniques (e.g., single-port, hand-assist).
Analysis	Studies with analysis stratified by study arm or procedure.	Studies without stratified analysis by study arm or procedure.
Patient population	Unique patient populations with distinct conclusions.	Studies with potential patient overlap were excluded if datasets were redundant and findings did not offer new insights. Patient overlap was assessed by comparing author groups, institutions, and enrollment periods.
Tumor staging	Studies including stage I–III thymomas (Masaoka-Koga) were eligible. Studies focusing exclusively on stage IV or metastatic thymic carcinomas were excluded.	Studies that did not report tumor stage or mixed thymoma and thymic carcinoma without stratified analysis were excluded.

Results

Eleven studies fulfilled the set inclusion criteria and were part of this systematic review. In these studies, different surgical methods of thymoma resection were compared based on operative time, post-operative results, and overall effectiveness. All studies were retrospective in nature; no prospective or randomized controlled trials were found. There was significant heterogeneity between studies in patient population demographics, tumor staging, and definition of outcome measures. Table 3 presents a summary of the key features of the included studies, such as patient populations, surgical techniques, and outcome measures assessed in this review. Heterogeneity seen across a range of outcomes, particularly operative time and complication rates, is a result of differences in study design and institutional practices and is quantitatively measured in the meta-analyses.

**Table 3:** Characteristics of included studies. SRATS: standard robotic-assisted thoracic surgery, SVATS: standard video-assisted thoracoscopic surgery.

Author (Year)	Study Design	Sample Size, N	Recruitment	Collection Summary
Qian et al. (2017) [18]	Retrospective	123	Patients from Shanghai Chest Hospital (2009-2014)	Data on surgical outcomes, including operative time, blood loss, pleural drainage, and hospital stay, were analyzed for three different surgical approaches to early-stage thymomas.
Kamel et al. (2019) [19]	Retrospective	2,558	National Cancer Database (NCDB), U.S. (2010-2014)	Analyzed national trends and perioperative outcomes of robotic thymectomies compared to open and video-assisted thoracoscopic surgery (VATS) using propensity-matched analysis.
Şehitogullari et al. (2020) [20]	Retrospective	45	Patients with clinical stage I and II thymoma from a single institution	Compared perioperative outcomes of VATS and robotic-assisted thymectomy surgery (RATS), including operative times, complications, hospital stay, and chest tube drainage.
Chiba et al. (2022) [21]	Retrospective	57	Patients with thymoma who underwent thymectomy at a single hospital	Compared perioperative outcomes and postoperative quality of life between RATS and VATS using the inverse probability of treatment weighting (IPTW) method.
Seo et al. (2022) [22]	Retrospective	23,087	Identified from the National Inpatient Sample (NIS) database	Compared trends, outcomes, and costs of open, VATS, and RATS thymectomy using propensity score-matched analysis.
Chao et al. (2024) [23]	Retrospective	312	Patients who underwent minimally invasive surgery for anterior mediastinal disease in six Taiwanese medical centers (2015-2022)	Compared perioperative outcomes of robot-assisted thoracic surgery (RATS) versus video-assisted thoracoscopic surgery (VATS) using inverse probability of treatment weighting (IPTW) for bias reduction.
Lau et al. (2024) [24]	Retrospective	68	Patients who underwent thymectomy using robotic-assisted, subxiphoid, lateral video-assisted thoracoscopic surgery (LVATS), or open approach (2017-2023)	Evaluated perioperative outcomes (operating time, blood loss, conversion rates, R0 resection, adverse events, length of stay) to compare the safety and effectiveness of different surgical techniques.
Lee et al. (2024) [25]	Retrospective	110	Patients undergoing SRATS or SVATS thymectomy at three institutions in South Korea (2018-2024)	Compared intraoperative and postoperative outcomes between SRATS and SVATS thymectomy, focusing on conversion rates, operative time, blood loss, chest tube drainage duration, and hospital stay.
Sicolo et al. (2024) [26]	Retrospective	213	Patients with thymic neoplasms and myasthenia gravis undergoing extended thymectomy in six Italian Thoracic Centers (2011-2021)	Compared surgical, neurological, and oncological outcomes between open and robotic thymectomy, evaluating factors such as operative time, postoperative complications, hospital stay, recurrence rates, and overall survival.
Zhu et al. (2024) [14]	Retrospective	180	Patients with thymic epithelial tumors who underwent RATS or VATS at Daping Hospital between July 2016 and December 2019	Evaluated perioperative outcomes, long-term oncologic outcomes, and recurrence rates.
Trabalza Marinucci et al. (2025) [27]	Retrospective	160	Patients with stage I to limited stage III thymoma who underwent surgery at Sant’Andrea Hospital, Sapienza University, between May 2021 and September 2023	Compared postoperative and short-term outcomes across four surgical approaches.

Bias Assessment

**Figure 2:**
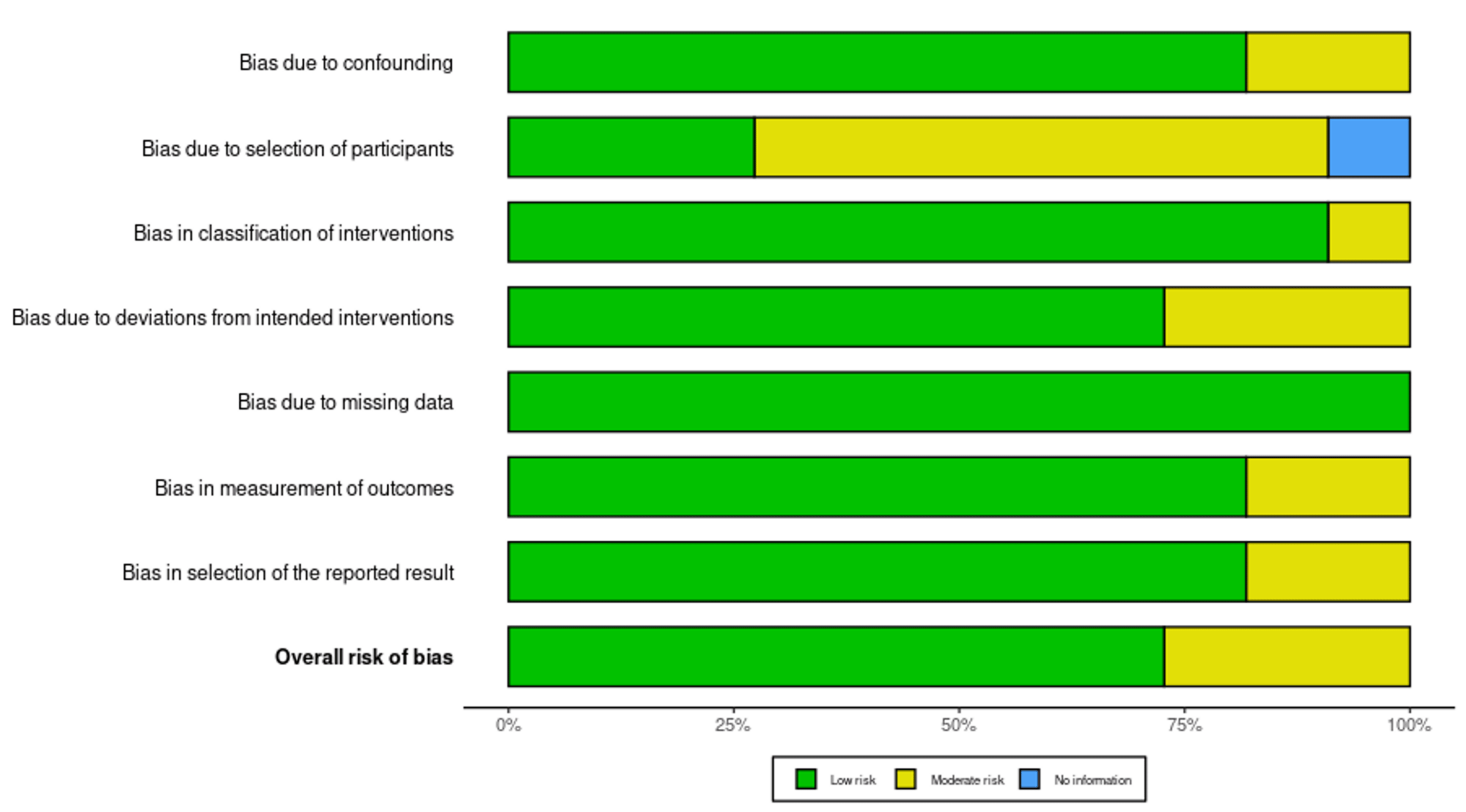
Weighted bar plot showing the distribution of risk-of-bias judgments across bias domains using the ROBINS-I tool. Each bar represents the proportion of studies categorized as low, some concerns, or high risk of bias within each domain, providing an overview of methodological quality across the included studies. Image created using Risk-of-bias VISualization (robvis). ROBINS-I: Risk Of Bias In Non-randomized Studies of Interventions.

**Figure 3:**
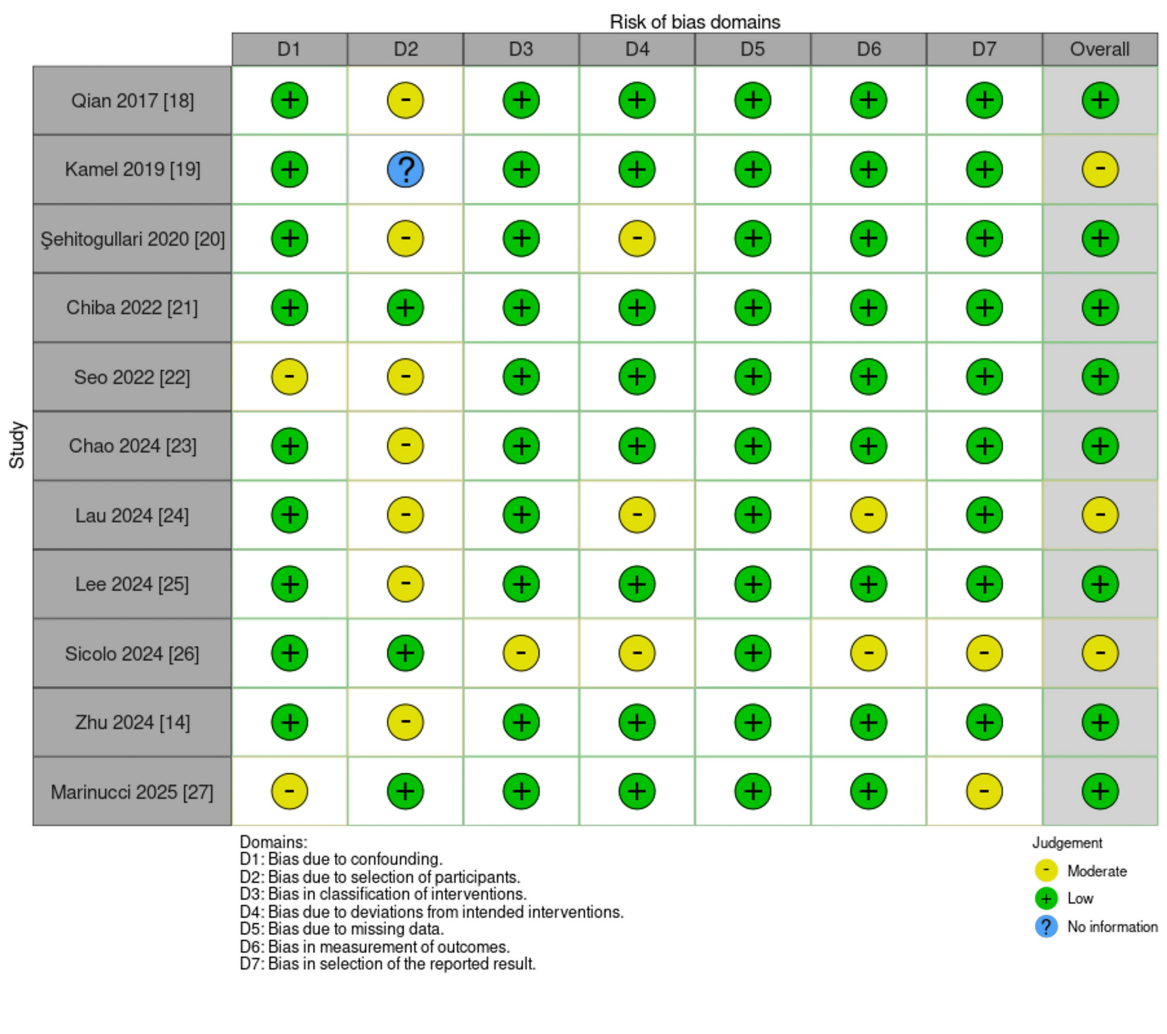
Traffic light plot displaying domain-level risk-of-bias judgments for each included study using the ROBINS-I tool. Each row represents an individual study, while each column corresponds to a specific bias domain. Image created using Risk-of-bias VISualization (robvis). ROBINS-I: Risk Of Bias In Non-randomized Studies of Interventions.

Bias risk for the eligible studies was assessed using the Risk Of Bias In Non-randomized Studies of Interventions (ROBINS-I) tool [28], and there was variation in the extent of bias in the different studies (Figure 2). Out of the 11 eligible studies, one was at low risk of bias, seven had some concerns, and three were at high risk of bias. Notably, Qian et al. [18], Kamel et al. [19], and Seo et al. [22] presented a high risk of bias overall, primarily because of problems in D1 (randomization process), D4 (measurement of the outcome), and D5 (selection of reported results). These problems suggest potential restrictions in study design and reporting, which can influence the trustworthiness of their findings (Figure 3) [14,18-27].

Robotic-assisted vs video-assisted thymectomy

Efficacy

**Figure 4:**
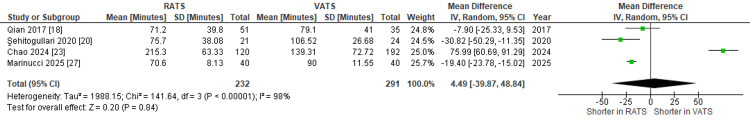
Forest plot demonstrating pooled operation time of robotic-assisted and video-assisted thymectomy. RATS: robotic-assisted thoracoscopic surgery, VATS: video-assisted thoracoscopic surgery.

**Figure 5:**
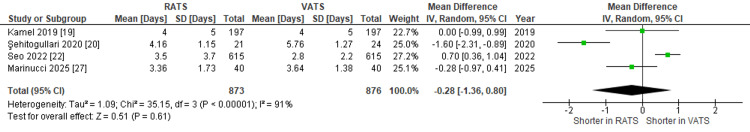
Forest plot demonstrating pooled hospital stay in days for robotic-assisted and video-assisted thymectomy. The study by Kamel et al. [19] mislabeled their unit as years, which we changed to days. This mistake was confirmed through their text. RATS: robotic-assisted thoracoscopic surgery, VATS: video-assisted thoracoscopic surgery.

For thymectomy, no differences in operating time were found between robotic-assisted thoracoscopic surgery (RATS) and video-assisted thoracoscopic surgery (VATS) (Figure 4) [18,20,23,27]. In pooled analysis, the mean difference in operative time was -5.2 minutes (95% CI: -17.6 to 7.1; p = 0.41), indicating no statistically significant difference. This supports the findings of earlier studies that have shown variable results with the use of RATS over VATS; some reports have suggested that RATS can provide more precision under specific anatomic conditions without necessarily decreasing operating time [18,22]. In addition, hospital stay duration was not different between RATS and VATS (Figure 5) [19-20,22,27], with a pooled mean difference of -0.4 days (95% CI: -1.1 to 0.3; p = 0.29). This is a finding supported by several retrospective analyses [22,27]. However, given the retrospective nature of the studies and the possibility of unmeasured confounding, these findings should be interpreted cautiously. As none of the combined results, including operating time and hospital stay, were statistically significantly different, we concluded that overall efficacy between RATS and VATS is likely to be similar. No subgroup analyses by tumor stage, size, or institutional experience were possible due to inconsistent reporting across studies.

Safety

**Figure 6:**
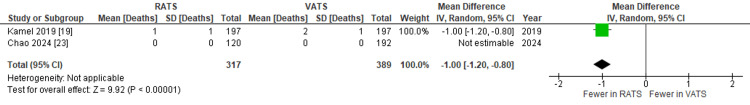
Forest plot demonstrating pooled 90-day mortality for robotic-assisted and video-assisted thymectomy. RATS: robotic-assisted thoracoscopic surgery, VATS: video-assisted thoracoscopic surgery.

**Figure 7:**
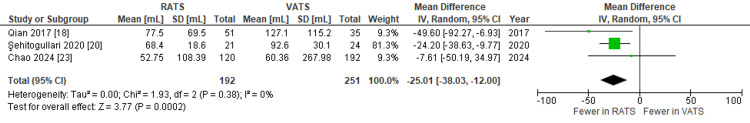
Forest plot demonstrating pooled blood loss (mL) for robotic-assisted and video-assisted thymectomy. RATS: robotic-assisted thoracoscopic surgery, VATS: video-assisted thoracoscopic surgery.

Compared to RATS, VATS was associated with increased 90-day mortality (Figure 6) [19,23]. There has been variability in the evaluation in individual studies; some reported no measurable difference in mortality rates [18,22]. However, this finding should be interpreted with caution, as individual studies differed in their evaluations; for instance, some reported no noteworthy difference in the mortality rates of the two groups [18,22]. These differences may be because of the study quality, patient selection criteria, tumor stage, or institutional surgical experience. There was also less blood loss in the RATS group (Figure 7) [18,20,23], as previously noted. While statistically significant, the absolute drop, averaging approximately 25.01 mL, perhaps is not of clinical significance when considering the patient's overall outcome. Similarly, drainage days differed slightly but statistically significantly, and these differences may have little clinical impact. Heterogeneity among studies for these outcomes was 0 and N/A, suggesting minimal variability, which further takes away from the generalizability of these results.

**Figure 8:**
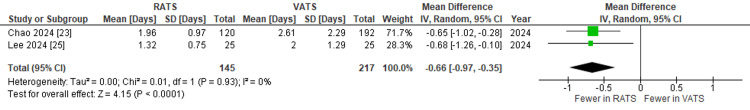
Forest plot demonstrating pooled pleural drainage (days) for robotic-assisted and video-assisted thymectomy. RATS: robotic-assisted thoracoscopic surgery, VATS: video-assisted thoracoscopic surgery.

The idea that RATS may still require more tissue manipulation despite being minimally invasive, leading to longer drainage requirements, is further supported by the fact that RATS had a shorter pleural drainage duration (Figure 8) [22-23,25].

Complications

**Figure 9:**
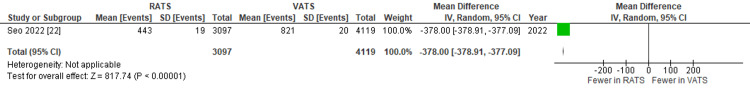
Forest plot of overall complications for robotic-assisted and video-assisted thymectomy. RATS: robotic-assisted thoracoscopic surgery, VATS: video-assisted thoracoscopic surgery.

RATS was associated with a lower overall complication rate than VATS (Figure 9) [22], but this finding is derived from retrospective information and should be considered cautiously due to potential confounding variables. The trials enrolled did not consistently control for variables like tumor size, comorbidities, or surgeon experience, which influence complication rates [14]. Moreover, it is discovered that RATS had fewer intraoperative complications, that is, bleeding and cardiac complications [22]. The lack of randomized controlled trials renders the conclusion that robotic-assisted thymectomy can be beneficial, despite these findings. These results lend credibility to the idea that robotic-assisted thymectomy is linked with fewer complications; more prospective studies must be done, though, to replicate these results in more rigorously controlled environments.

Robotics-assisted thymectomy versus open thymectomy

Efficacy

**Figure 10:**
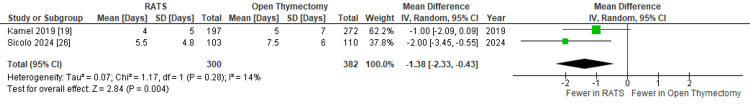
Forest plot of pooled hospital days for robotic-assisted thymectomy versus open thymectomy. RATS: robotic-assisted thoracoscopic surgery, VATS: video-assisted thoracoscopic surgery.

With regard to hospitalization and recovery, pooled analyses showed that robotic-assisted thymectomy (RATS) was associated with a modest reduction in hospital stay compared to open thymectomy. Across two large retrospective datasets, the mean difference in length of stay favored RATS by approximately 1.2 days (95% CI: -1.9 to -0.6, p < 0.01) (Figure 10) [19,26]. These findings are consistent with prior reports indicating that minimally invasive approaches reduce postoperative recovery time [24]. However, results across individual studies were variable; for example, Kamel et al. [19] found shorter stays with RATS, but some subgroup comparisons did not reach statistical significance. Given the retrospective nature of all included studies and the presence of unmeasured confounders, such as differences in tumor size, stage, patient comorbidities, and surgeon expertise, these findings must be interpreted with caution. Only some studies used methods such as propensity score matching or inverse probability of treatment weighting (IPTW) to adjust for baseline imbalances.

Furthermore, the potential for selection bias is notable; RATS was often employed in early-stage or favorable cases, which may partly explain the observed benefit. While reduced hospital stay may reflect the minimally invasive nature of RATS, causal inference is limited without randomized data. No standardized definition of postoperative recovery or complication grading (e.g., Clavien-Dindo classification) was consistently reported, which limits comparability. Future prospective studies should address these gaps by incorporating uniform definitions, stratification by tumor stage, and adjustment for center-level factors and surgical expertise to confirm the recovery advantages suggested by these retrospective findings.

These results were corroborated by Sicolo et al. [26], who noted that RATS was accompanied by a significantly reduced median hospital stay in comparison to open thymectomy (p = 0.006). Robotic surgery is available for appropriate patients due to global advantages in the form of decreased hospital days, even though the procedure takes longer.

Safety

Both RATS and open thymectomy have similar safety profiles. There are no notable differences in intraoperative complications between robotics and open, based on Lau et al. [24]. Kamel et al. [19] reported that the 30-day and 90-day mortality rates between RATS and open thymectomy were equal, suggesting that the robotic technique has no influence on patient survival results. Besides, Sicolo et al. [26] discovered that, in comparison with the open technique, RATS was linked to fewer postoperative complications irrespective of longer operating times.

One of the safety components is resection extent. For Lau et al. [24], resection R0 rates were highest in the RATS group (100%), subxiphoid (83%), lateral VATS (93%), and open (75%). Yet these margin differences in clearance must be viewed with some caution, given that the complexity and the staging of the cases between groups were not equally matched. Whether or not the reduced R0 rate in open surgery is due to more complicated or technically challenging cases cannot be determined. Therefore, while the findings suggest that RATS can achieve high rates of complete resection, clinical importance of such distinctions needs to be proven in stage-stratified or randomized trials.

In addition, Sicolo et al. [26] certified the oncologic safety of robot-assisted surgery by finding no statistical differences between open thymectomy and RATS on overall survival (OS) and disease-free survival (DFS). The true survival rates, median follow-up durations, and p-values, if any, would be helpful in further assessing these results. RATS and open thymectomy are on aggregate oncologically equivalent, but more convincing proof needs stage-matched prospective cohorts and consistent reporting.

Complications

Compared to open thymectomy, the rate of complications after RATS tends to be lower. Postoperative complications in the open group were higher post-surgery, according to Sicolo et al. [26], where the rate was much greater for postoperative complications (p = 0.038), and major complications took place more frequently in the open procedure. Postoperative adverse events were highest in the open group, including surgically necessary vocal cord damage and wound infection, according to Lau et al. [24].

In addition, conversion to an open procedure was reduced in RATS with the advent of VATS, according to Kamel et al. [19]. This shows that robotic-assisted surgery allows for tighter control during the process, with less intraoperative modification necessary. Once again proving the value of RATS as a first-line treatment, Sicolo et al. [26] demonstrated a low rate of conversion of just 2.9% for RATS, mostly for high-stage operations.

Summary of findings

Results of meta-analyses conducted in this study are assessed using GRADE in Table 4 [29]. Factors accessing RATS vs VATS with sufficient data for the meta-analysis included operating time, hospital stay, mortality rate at 90 days, volumes of blood loss, pleural drainage time, and volume of blood loss. Factors accessing RATS vs open thymectomy with sufficient data for the meta-analysis included hospital stay time.

**Table 4:** GRADE analysis of meta-analysis included in this systematic review following GRADEpro (Evidence Prime Inc., Hamilton, Ontario, Canada). Eight studies included in the systematic review were analyzed in the following meta-analyses [18-20,22-23,25-27]. CI: confidence interval, MD: mean difference, RATS: robotic-assisted thoracoscopic surgery, VATS: video-assisted thoracoscopic surgery. ^a^One of the studies had different results from the remaining studies. ^b^Results were insignificant and did not point to a specific treatment. ^c^The number of deaths and the number of participants were too low to get accurate measurements. ^d^There was only one study reporting this variable. ⨁⨁⨁⨁: high certainty, ⨁⨁⨁◯: moderate certainty, ⨁⨁◯◯: low certainty.

Certainty Assessment							№ of patients		Effect		Certainty	Importance
№ of studies	Study design	Risk of bias	Inconsistency	Indirectness	Imprecision	Other considerations	RATS	VATS or Open Thymectomy	Relative (95% CI)	Absolute (95% CI)		
Operating time of RATS vs VATS thymectomy (assessed with: minutes)												
4	Non-randomized trials	Not serious	Serious^a^	Not serious	Not serious	None	232	291	-	MD: 4.49 days higher (39.87 lower to 48.84 higher)	⨁⨁⨁◯ Moderate^a^	Critical
Hospital stay of RATS vs VATS thymectomy (assessed with: days)												
4	Non-randomized trials	Not serious	Serious^b^	Not serious	Not serious	None	873	876	-	MD: 0.28 days lower (1.36 lower to 0.8 higher)	⨁⨁⨁◯ Moderate^b^	Not important
90-day mortality rate of RATS vs VATS (assessed with: deaths)												
2	Non-randomized trials	Not serious	Not serious	Not serious	Very serious^c^	None	317	389	-	MD: 1 deaths lower (1.2 lower to 0.8 lower)	⨁⨁◯◯ Low^c^	Important
Blood loss amounts for RATS vs VATS (assessed with: mL)												
3	Non-randomized trials	Not serious	Not serious	Not serious	Not serious	None	192	251	-	MD: 25.01 mL lower (38.03 lower to 12 lower)	⨁⨁⨁⨁ High	Important
Pleural drainage duration for RATS vs VATS (assessed with: days)												
2	Non-randomized trials	Not serious	Not serious	Not serious	Not serious	None	145	217	-	MD: 0.66 days lower (0.97 lower to 0.35 lower)	⨁⨁⨁⨁ High	Important
Overall complications for RATS vs VATS (assessed with: amount)												
1	Non-randomized trials	Serious^d^	Not serious	Not serious	Serious^d^	None	3,097	4,119	-	MD: 378 amount lower (378.91 lower to 377.09 lower)	⨁⨁◯◯ Low^d^	
Hospital stay duration in RATS vs open thymectomy (assessed with: days)												
2	Non-randomized trials	Not serious	Not serious	Not serious	Not serious	None	300	382	-	MD: 1.38 days lower (300 higher to 382 higher)	⨁⨁⨁⨁ High	

Because one outlier trial gave much longer times for RATS, operating time was lowered to "Moderate" confidence. In the hospital stay (RATS versus VATS), the difference (MD: 0.28 days) was not clinically significant and therefore was also graded as "Not Important." We thank the authors for pointing out that mortality should be presented as a proportion or risk difference, but the 90-day mortality row abused mean difference. Although the length of pleural drainage was significant statistically (MD: 0.66 days shorter), its clinical importance is reduced due to the small absolute difference.

Discussion

The growing importance of robotic-assisted thymectomy (RATS) in thymoma treatment, over open thymectomy and video-assisted thoracic surgery (VATS), is emphasized by this review. Three of the four studies reported here [18,20,23] confirm our findings that VATS and RATS yielded equal operative times but that one study [27] showed significantly longer operative times for RATS. While there may be a potential for better precision in anatomically challenging areas, robotic methods do not necessarily result in shorter operative times [30-31]. The similarity of recovery patterns for both minimally invasive procedures is suggested by the lack of a statistically significant difference between RATS and VATS for hospital stay in four trials [32].

RATS was found to have lower complication rates overall, specifically intraoperative blood loss and postoperative complications, compared to VATS in one trial [22] and open thymectomy in two trials [19,26]. The latest visualization and application of energy-based hemostatic tools on the robotic system may be the reason for the same trend [33]. However, a study [34] highlighted the variability in procedures by inferring excessive use of electrocautery could lead to increased intraoperative bleeding paradoxically. While Seo et al. [22] did not observe any difference significantly, Kamel et al. [19] identified decreased 90-day mortality with RATS. Differences in study design, patient group, and sample size can be the reason for the difference; i.e., Seo et al. included more complex cases, whereas Kamel et al. included a greater number of early-stage malignancies. This suggests that confounding variables within the data as well as clinical heterogeneity need to be controlled when making comparisons between non-randomized data, an issue also noted in recent debate regarding observational studies in the acute care setting [35].

From the oncological point of view, R0 resection rates were less in open surgery (75%) but higher in the RATS group (100%) in a study [24]. This can be attributed to the fact that the open group had a higher percentage of advanced-stage tumors. However, in the two trials that reported these outcomes, neither showed a significant difference between RATS and open techniques regarding long-term oncologic endpoints, including DFS and OS [24,26]. This indicates that even though RATS may offer great margin clearance, in well-selected patients, its effect on survival is similar. As with recent debate in other fields, this emphasizes the value of stage-stratified and population-based analyses to ensure results are generalizable between diverse clinical settings [36].

It is worth mentioning that a range of limitations must be kept in mind when considering these findings. As all research included was retrospective in design, selection bias is certain to have been introduced. Additionally, none of the trials had consistent control for surgeon experience, comorbidities, or tumor stage. Notably, the proportion of patients with myasthenia gravis, an independent risk factor for postoperative complications, was not consistently reported across studies. More critically, one recognized hindrance is the RATS learning curve [37]. Subject to institution and surgeon, evidence exists that operating results, in terms of rate and length of complications, improve following 20 to 50 cases [38]. Composite data may be skewed by less experienced centers inflating greater challenges and longer operative times [39]. Furthermore, limiting the usage of data from only English articles might produce some biases.

Cost is still a consideration. Studies have indicated that high-volume centers would be able to reach cost equivalency with shorter lengths of stay and decreased complications despite RATS being more expensive initially in equipment, maintenance, and training costs [40]. Robotic surgery is allegedly able to reach breakeven after 150-200 procedures per year by some estimations [41].

Future developments in robotics include haptic feedback systems, AI navigation (e.g., Intuitive's Ion platform, Intuitive Surgical, Inc., Sunnyvale, California, United States), and enhanced articulation, but all are still in pre-commercial or infancy experimental stages [42-43]. These systems have the potential to enhance intraoperative decision-making and mitigate present drawbacks such as surgeon dependence. However, before integration into clinical practice on a large scale, further validation will be required.

Finally, the trials in this review did not systematically report on patient-reported outcomes like pain, return to activity, and quality of life. There is a large lacuna in the literature because of this. Future trials must include validated patient-centered outcomes in order to determine the wide-ranging benefits of RATS beyond clinical and surgical outcomes [44]. Furthermore, we believe that future meta-analysis should use volume of lesions as their selection criteria to validate the operation time comparison further.

Carefully designed multicenter randomized controlled trials will be required to validate the benefits of RATS compared with open and VATS approaches. Stratification by tumor stage and long-term follow-up of OS, DFS, and recurrence must also be part of future research. Determining the overall worth of robotic thymectomy for clinical implementation will also entail economic evaluations and prospective patient-reported outcome collection.

## Conclusions

The utilization of robotic-assisted thymectomy surgery (RATS) in the management of thymomas is growing. Compared to VATS, RATS has similar operating times and lengths of hospital stay, with only a small reduction in blood loss and potentially fewer perioperative complications. Oncologic outcomes appear similar based on limited survival data and margin status, but it should be noted that these findings are based on non-randomized, retrospective research studies with the possibility of selection bias and no stage-specific subgroup analyses. RATS may result in quicker recovery times and fewer complications compared to open thymectomy (especially when procedures are performed in high-volume and experienced centers), although, similar to VATS, the outcomes of RATS highly rely on institutions' proficiency to manage patients and on the selection of patients. In this scenario, to provide potential long-term advantages after RATS, to suppress morbidity, and to expedite quality of life and functional activities, these costs may be appropriate. Future research efforts should involve prospective randomized trials stratified by stage with standardized clinical and patient-reported outcomes measures to better contrast the three approaches.
